# A Substitute for Iodoform

**Published:** 1915-11

**Authors:** 


**A Substitute for Iodoform.** Mouchet and Malbec found iodoform of value in the treatment of infected, especially of gangrenous wounds. The fetor disappeared, and the foul scab covering the surface gave place to healthy granulations. The extremely disagreeable smell of iodoform is an objection to its use, and an efficient substitute was discovered in quiniodol, which smells good, is just as powerfully antiseptic and equally keratoplastic. The iodine is in as good a state of dilution as in the liquid form. Quiniodol is cheaper than iodoform. The method of preparation is as follows: 1. Quiniodol 5 per cent. a. Dissolve 5 gr. of iodine in 100 c.c. of ether. b. Add 100 gr. of powdered red cinchona. c. Triturate the mass until the ether has evaporated completely. d. Pass the powder so obtained through a fine sieve. 2. Quiniodol 10 per cent. 10 gr. of iodine are dissolved in 200 c. c. of ether and triturated with 100 gr. of powdered cinchona. The 10 per cent strength is used for gangrenous wounds, the 5 per cent for sluggish and slightly infected wounds. —Paris Medical.

